# The uPA System Differentially Alters Fibroblast Fate and Profibrotic Ability in Skin Fibrosis

**DOI:** 10.3389/fimmu.2022.845956

**Published:** 2022-03-16

**Authors:** Ming-Li Zou, Ying-Ying Teng, Zhong-hua Chen, Si-Yu Liu, Yuan Jia, Kai-Wen Zhang, Jun-Jie Wu, Zheng-Dong Yuan, Xiao-Yu Tang, Shun Yu, Jun-Xing Ye, Xia Li, Xiao-Jin Zhou, Feng-Lai Yuan

**Affiliations:** ^1^ Institute of Integrated Traditional Chinese and Western Medicine, The Affiliated Hospital of Jiangnan University, Wuxi, China; ^2^ Wuxi Hospital of Integrated Chinese and Western Medicine, Nanjing University of Chinese Medicine, Wuxi, China; ^3^ Institute of Integrated Traditional Chinese and Western Medicine, The Third Hospital Affiliated to Nantong University, Wuxi, China

**Keywords:** skin fibrosis, scleroderma, uPA system, systemic sclerosis, fibroblast

## Abstract

Skin fibrosis is a common pathological feature of various diseases, and few treatment strategies are available because of the molecular pathogenesis is poorly understood. The urokinase-type plasminogen activator (uPA) system is the major serine protease system, and its components uPA, urokinase plasminogen activator receptor (uPAR) and plasminogen activator inhibitor-1(PAI-1) are widely upregulated in fibrotic diseases, including hypertrophic scars, keloids, and scleroderma. Here, we found that the successful binding of uPA and uPAR activates the downstream peroxisome proliferator-activated receptor (PPAR) signalling pathway to reduce the proliferation, migration, and contraction of disease-derived fibroblasts, contributing to the alleviation of skin fibrosis. However, increased or robust upregulation of the inhibitor PAI-1 inhibits these effects, suggesting of the involvement of PAI-1 in skin fibrosis. Subsequent *in vivo* studies showed that uPAR inhibitors increased skin fibrosis in mouse models, while uPA agonists and PAI-1 inhibitors reversed these effects. Our findings demonstrate a novel role for the uPA system and highlights its relationships with skin fibrosis, thereby suggesting new therapeutic approaches targeting the uPA system.

## Introduction

Fibrosis is a common process characterized by excessive extracellular matrix (ECM) accumulation after inflammatory injury (1). Fibrosis is involved in the pathogenesis of most diseases of the heart, liver, kidney, lung, skin and other tissues. With the increasing attention and detection of the process, tissue fibrosis has become one of the main causes of disability and deaths due to many diseases (2). As a critical barrier between inside and outside of the body, the skin works continuously to protect animals against pathogen and injuries. These functions also make the skin an easily damaged organ. Several common types of skin injuries caused by diseases, such as hypertrophic scars, keloids and scleroderma, are known to be accompanied by skin fibrosis characterized by the excessive healing of tissues (3). Currently there is no effective therapy for this pathological alteration. Further investigation on the mechanisms of the fibrotic process is required for better management of skin fibrosis.

An accumulating body of evidence supports the crucial role of the urokinase-type plasminogen activator (uPA) system for the development of several fibrotic diseases. Horowitz et al. suggested that restoring intrapulmonary plasminogen activity could alleviate the lung fibrogenesis and promote the resolution of established lung fibrosis, indicating an positive impact by uPA (4). It was also reported that the urokinase plasminogen activator receptor (uPAR) deficiency could accelerate the renal fibrosis in obstructive nephropathy (5). Indeed, transplantation of uPA gene-modified bone marrow-derived mesenchymal stem cells has been proposed as a potential approach to treat liver fibrosis and ameliorate liver function (6).

The uPA system, a major system of serine proteases, is composed of uPA, uPAR, plasminogen activator inhibitor-1 (PAI-1) and plasminogen activator inhibitor-2 (PAI-2) (7). uPAR is a multifunctional receptor anchored to the surface of various cell types including neutrophils, monocytes, fibroblasts, endothelial cells, and a variety of malignant cells. As a well-characterized receptor, uPAR functions by binding to uPA and pro-uPA (8). uPAR catalyses the conversion of pro-uPA to uPA, leading to the conversion of inactive plasminogen to active plasmin, the activation of the fibrinolytic system, and finally the degradation of various matrix proteins (9, 10). In addition, uPAR affects multiple signalling pathways by interacting with other transmembrane proteins, which modulates the transcription and expression of downstream signalling proteins, and ultimately the biological properties of cells (11–14). Since unrestrained proteolytic activities are hazardous to cells, plasmin activity is tightly controlled by the plasminogen activator inhibitors PAI-1 and PAI-2. PAI-1 represents the primary physiological inhibitor of uPA and regulates the proteolytic activity of uPA directly *via* its serine proteinase activity and indirectly by regulating the levels of the uPA-uPAR complex through promoting endocytosis of the complex (15, 16). However, the exact function of the uPA system in the development of skin fibrosis and the underlying mechanism remain largely unclear.

This study focuses on how the expression of uPA, uPAR and PAI-1 may regulate the proliferation, migration, and contractility of fibroblasts during the development of skin fibrosis in animal models and human. More findings in this area will not only enrich our knowledge on the uPA system, but also shed lights on targeted therapy against skin fibrosis.

## Results

### uPA, uPAR and PAI-1 Are Upregulated in Fibrotic Tissues

**Figure 1:**
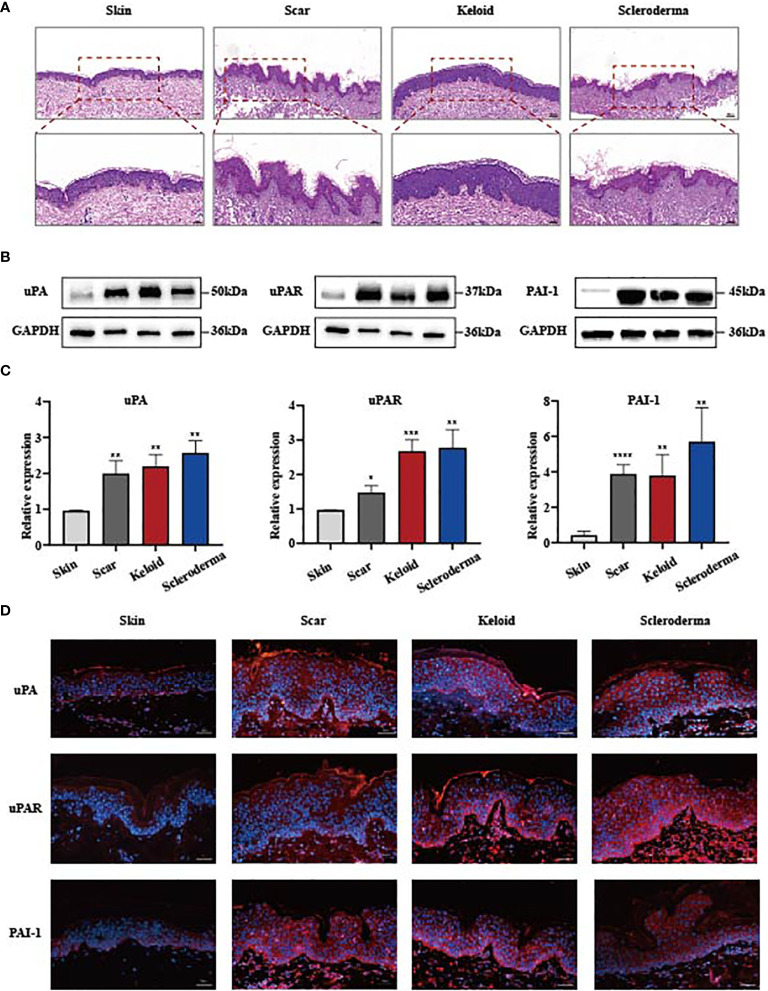
uPA, uPAR and PAI-1 are frequently upregulated in skin fibrosis. **(A)** Haematoxylin and eosin staining of human normal and fibrotic tissues. Scale bars, 100 μm, 50μm. **(B, C)** The protein level of uPA, uPAR and PAI-1 in normal and fibrotic tissues analyzed by the western blot assay. GAPDH was the internal reference. Hypertrophic scars, n=5; keloids, n=4; systemic sclerosis, n=7. **(D)** Immunofluorescence staining of uPA, uPAR and PAI-1 in normal and fibrotic tissues. Scale bars, 100 μm. *P < 0.05, **P < 0.01, ***P < 0.001, and ****P < 0.0001 when compared between the denoted groups.

To investigate the relationship between the uPA, uPAR and PAI-1 expression and skin fibrosis, we compared the protein expression patterns in normal and three different types of fibrotic samples including hypertrophic scars, keloids, and scleroderma tissues. Haematoxylin and eosin (HE) staining of these tissues verified a significant increase in the thickness of the dermal layers in these fibrotic tissues (**Figure 1A**). The expression of uPA, uPAR and PAI-1 proteins in normal skin was examined by Western blotting. While normal skin samples contained low levels of these proteins, fibrotic skin tissues expressed significantly higher levels (**Figures 1B, C**). Immunofluorescence staining confirmed the dramatic increase of these proteins in the fibrotic skin tissues (**Figure 1D**). The strong clinical association between upregulated uPA, uPAR and PAI-1 expression and skin fibrotic changes provide a solid basis to further investigate their functions and pathways using cell culture and animal models.

### Knockdown of uPAR Promotes Fibrosis Progression *In Vitro*

**Figure 2:**
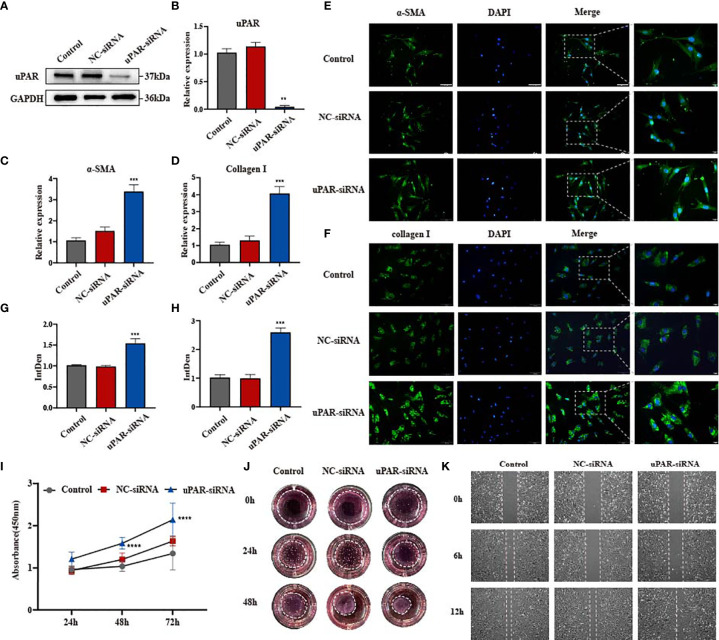
Knockdown of uPAR promotes fibrosis progression *in vitro*. **(A, B)** The knockdown efficiency of uPAR-siRNA in disease-derived fibroblasts was confirmed by western blot and qPCR. **(C, D)** mRNA level of α-SMA and type I collagen in three groups of cells. **(E–H)** Immunofluorescence staining of α-SMA and type I collagen in three groups of cells and their integrated density. scale bars, 100 μm, 20μm. **(I)** The proliferation ability after knocking down uPAR was evaluated by the cell counting kit 8. **(J)** Effect of knocking down uPAR on the contractility of fibroblasts. **(K)** The migration ability of fibroblasts after knocking down uPAR was evaluated by wound-healing assay. **P < 0.01, ***P < 0.001, and ****P < 0.0001 when compared between the denoted groups.

With uPA, uPAR and PAI-1 all being widely upregulated in fibrotic tissues, we wondered which proteins may play the dominant function in skin fibrogenesis. We examined the significance of uPAR with the use of uPAR-small interfering RNA (siRNA) and primary culture of disease-derived fibroblast. After 24-48 h of cell transfection, the expression of uPAR was effectively knocked down as shown by qPCR and Western blotting, and the knockdown was effective at 48 h than that was at 24 h (**Figures 2A, B** and **Supplementary Figure 2B**). Previous studies have shown that the activation of ECM-producing myofibroblast, as evidenced by increased expression of myofibroblast markers such as alpha smooth muscle actin (α-SMA) and type I collagen, is a common feature for various fibrotic diseases (17–19). We conducted immunofluorescence analysis on α-SMA and type I collagen, and the results revealed that these proteins were upregulated in fibroblasts after uPAR knockdown (**Figures 2E–H**). The results of qPCR on α-SMA and type I collagen mRNA levels were consistent with those from immunofluorescence on their protein levels (**Figures 2C, D**). These observations indicated that downregulation of uPAR could lead to the aggravation of fibrosis.

Since fibroblast functions are closely related to the development of fibrosis, we examined changes in these cells’ proliferation, migration and contraction following uPAR knockdown. A significant increase in the growth rate was observed in the uPAR-siRNA group at both 24 h and 72 h, suggesting that uPAR knockdown accelerated fibroblast proliferation (**Figure 2I**). Subsequently, the migratory capacity were measured in scratch wound assays. A fast cell migration into the wound area and accelerated wound closure were observed in the uPAR-siRNA group when compared to the control group (**Figure 2K**, **Supplementary Figure 2F**). We performed the collagen gel contraction assay to measure the fibroblast contractility, a key factor for scar formation (20). Compared with the control group, cells from uPAR knockdown group exhibited an accelerated contraction in a collagen lattice from 24 to 48 h post-transfection (**Figure 2J** and **Supplementary Figure 2D**), suggesting that reduction of uPAR levels increased the contractility of human dermal fibroblasts. The above results strongly suggested that the knockdown of uPAR may contribute to the progression of dermal fibrosis likely by increasing fibroblast proliferation, migration and contractibility. It is consisted with the function of uPAR being the receptor of plasminogen activator, who can bind to uPA and activate the fibrinolytic system, together with degradating matrix proteins. However, we noticed that both the expression of uPAR and uPA is increased in fibrotic tissues in **Figure 1**, which seems to be contradictory. What makes uPA and uPAR fail to perform their functions? We noticed the inhibitor of plasminogen activator PAI-1.The expression of PAI-1 in fibrotic tissues is also increased and is much higher than uPA and uPAR. Would it possible that the existence of PAI-1 counteract the effect of plasminogen activator?

### Knockdown of PAI-1 Significantly Inhibits Fibrosis Progression *In Vitro*

**Figure 3:**
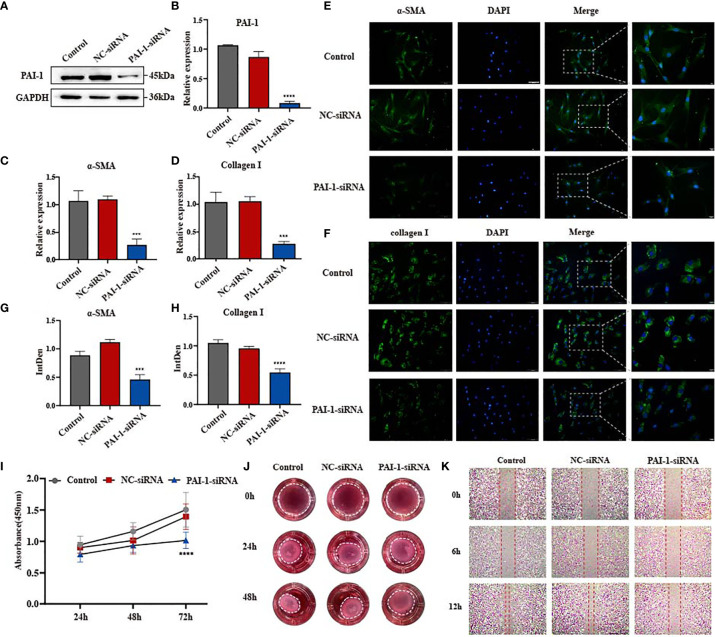
Knockdown of PAI-1 inhibits fibrosis progression *in vitro.* **(A, B)** The knockdown efficiency of PAI-1-siRNA in disease-derived fibroblasts was confirmed by western blot and qPCR. **(C, D)** mRNA level of α-SMA and type I collagen in three groups of cells. **(E–H)** Immunofluorescence staining of α-SMA and type I collagen in three groups of cells and their integrated density. scale bars, 100 μm, 20μm. **(I)** The proliferation ability after knocking down PAI-1 was evaluated by the cell counting kit 8. **(J)** Effect of knocking down PAI-1 on the contractility of fibroblasts. **(K)** The migration ability of fibroblasts after knocking down PAI-1 was evaluated by wound-healing assay. ***P < 0.001, and ****P < 0.0001 when compared between the denoted groups.

Based on the contradiction of uPAR overexpression and uPA-uPAR binding on the progression of skin fibrosis, we hypothesize that the presence of the inhibitor PAI-1 may promote skin fibrosis. PAI-1 has been frequently reported to play an important role in regulating fibrotic diseases such as pulmonary fibrosis and renal fibrosis (21–23). Considering the coincident upregulation of uPA, uPAR and PAI-1 in skin fibrosis, we focused on the specific role of PAI-1 in fibroblasts. Similarly, siRNA was used to knock down the expression of PAI-1 in disease-derived fibroblasts (**Figures 3A, B** and **Supplementary Figure 2C**). The immunofluorescence results suggested that α-SMA and type I collagen were obviously inhibited in PAI-1-knockdown cell lines (**Figures 3E–H**), which was further validated by qPCR on the mRNA level (**Figures 3C, D**). In cell function assays, PAI-1 knockdown significantly inhibited the proliferation of fibroblasts (**Figure 3I**). In addition, the scratch wound assay results suggested that the migratory capacities of myofibroblasts were decreased by knocking down PAI-1, suggesting its potential to promote tissue repair (**Figure 3K**). A collagen gel contraction assay was also used to investigate contractility, and the results indicated that PAI-1 knockdown significantly repressed the contraction of fibroblasts (**Figure 3J**). These results suggest that uPAR and PAI-1 may exert opposite activities in skin fibrosis. In summary, uPA together with uPAR promote the regression of fibrosis, while PAI-1 inhibits these effects.

### Local Inhibition of PAI-1 or the Addition of uPA Attenuates Bleomycin-Induced Skin Fibrosis *In Vivo*

**Figure 4:**
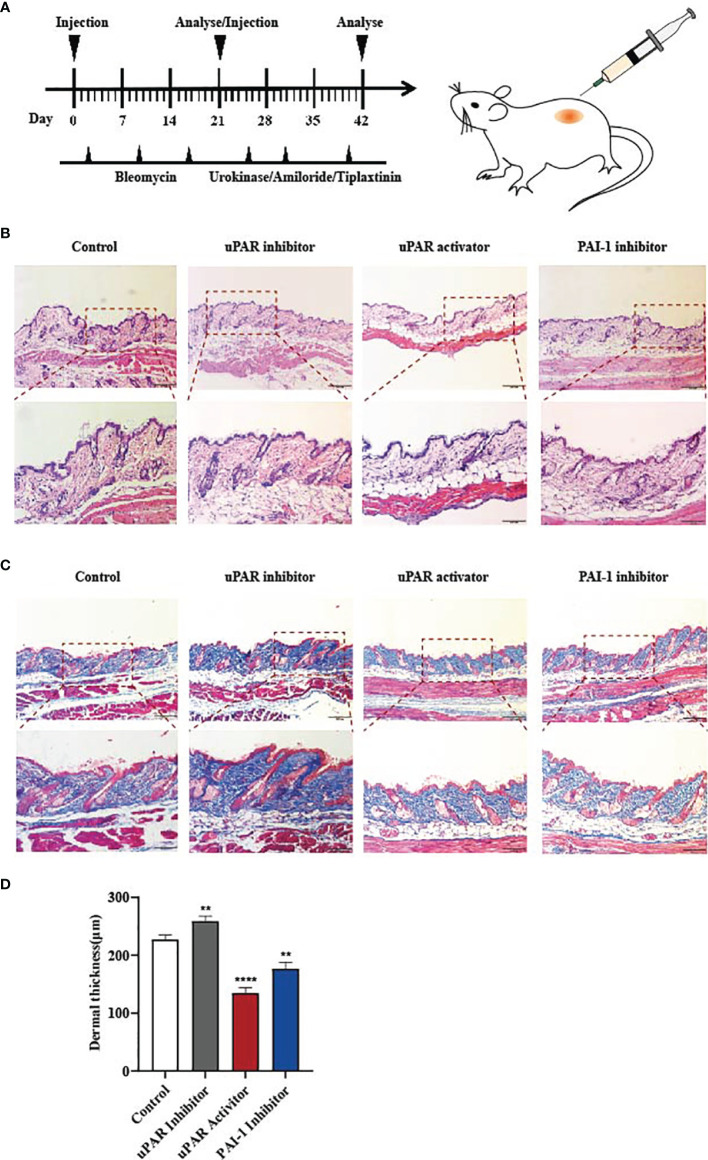
Inhibition of PAI-1 and the addition of uPA attenuate skin fibrosis *in vivo.* **(A)** Schematic overview of experimental design for **(B, C)**. **(B)** uPAR agonist and PAI-1 inhibitor attenuate skin fibrosis, while uPAR inhibitor aggravate skin fibrosis. n = 5. Scale bar, 200 μm, 100 μm. **(C)** Masson staining of tissues at 21 days after drug administration. n = 5. Scale bar, 200 μm, 100 μm. **(D)** Quantification of dermal thickness (μm). **P < 0.01 and ****P < 0.0001 when compared between the denoted groups.

**Figure 5:**
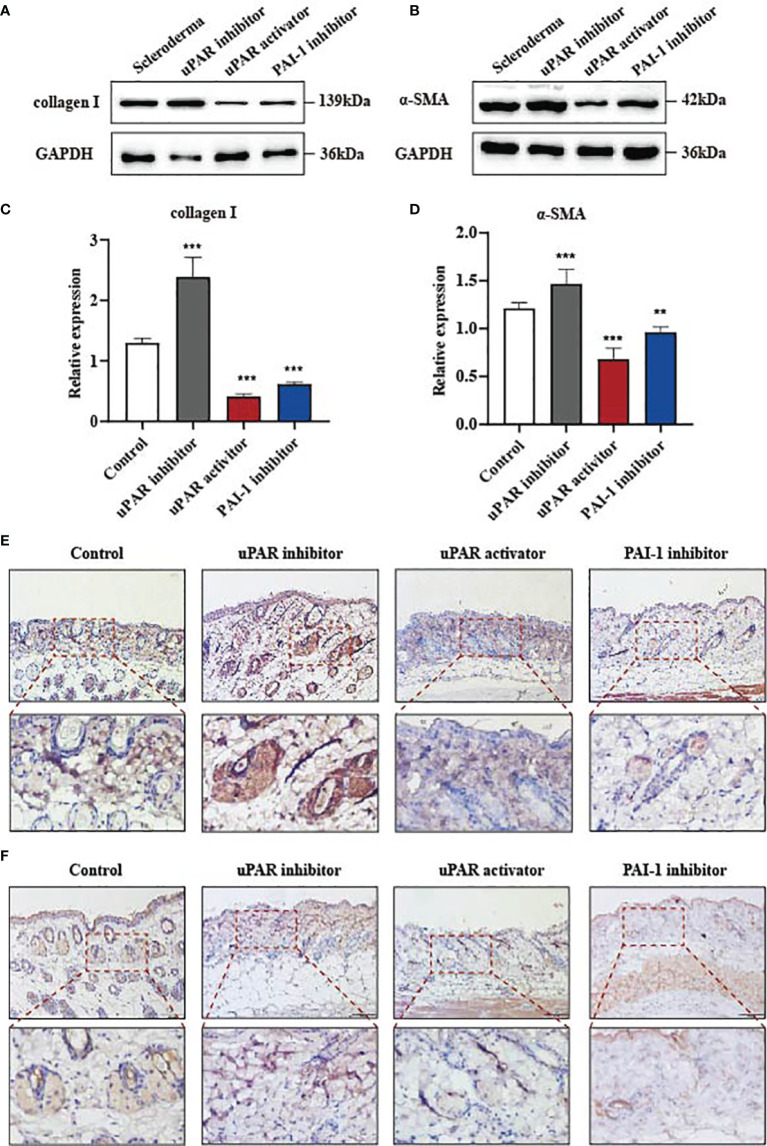
Inhibition of PAI-1 and the addition of uPA inhibit the expression of fibrosis associated proteins *in vivo*. **(A, B)** The proteins of α-SMA and type I collagen were detected from skins of mouse models by western blot assay. **(C, D)** Quantification of ** (A, B)**. **(E)** Skin tissues were immunohistochemically stained for type I collagen. Scale bar, 100 mm. **(F)** Skin tissues were immunohistochemically stained for α-SMA. Scale bar, 100 mm. **P < 0.01 and ***P < 0.001 when compared between the denoted groups.

Our data indicate that the reduction of PAI-1 or the increase of uPA might be a valuable therapeutic approach for treating skin fibrosis. Therefore, we used a mouse model of bleomycin-induced skin fibrosis to explore the functional significance of uPA, uPAR and PAI-1 *in vivo*. Bleomycin or phosphate-buffered saline (PBS) was injected subcutaneously into the mice daily for 3 weeks (**Figure 4A**). After the establishment of the SSc mouse model (**Supplemental Figure 3A**), the mice were divided into four groups and treated with PBS, a uPAR inhibitor, a uPAR agonist, or a PAI-1 inhibitor daily for an additional 3 weeks. HE and Masson’s trichrome staining revealed that treatment with uPAR inhibitor increased dermal thickening and collagen deposition, while treatment with the uPAR agonist or PAI-1 inhibitor significantly attenuated dermal thickness (**Figures 4B–D**). Additionally, we also noticed the decrease of α-SMA and type I collagen in mice treated with the uPAR agonist and PAI-1 inhibitor by western blotting and qPCR, which is consistent with the results of immunohistochemically staining (**Figures 5A–F**). All above indicated the decrease level of skin fibrosis as well as collagen deposition in uPAR agonist and PAI-1 inhibitor treated mice.

### uPA-uPAR Regulates the PPAR Signalling Pathway

**Figure 6:**
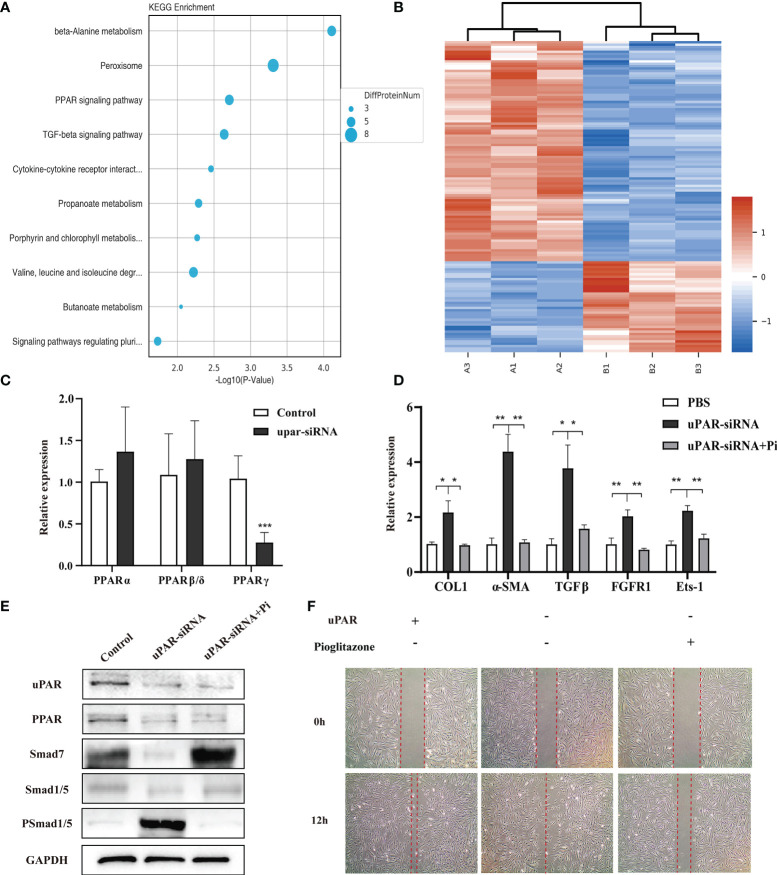
uPA-uPAR regulates the PPAR signalling pathway. **(A)** KEGG pathway enrichment analysis. **(B)** Cluster analysis of the different protein expression between fibroblasts of uPAR knockdown and control groups. **(C)** mRNA levels of PPARα, PPARβ, and PPARγ in uPAR-siRNA treated fibroblasts and control groups. **(D)** PPARγ agonist reversed the effects of silencing uPAR on the expression of fibrotic factors in fibroblasts. qRT-PCR assay was used to analyze the mRNA levels of COL1, α-SMA, TGFβ, FGFR1 and Ets-1. **(E)** Analysis of signaling pathway related proteins by western blot. **(F)** PPARγ agonist reversed the effects of silencing uPAR on cell migration. *P < 0.05, **P < 0.01 and ***P < 0.001 when compared between the denoted groups.

To explore the molecular mechanisms underlying the regulatory effect of the uPA system on skin fibrosis, the proteomic analysis was performed on cells in the uPAR-siRNA group and control group. The results showed that a total of 4655 proteins were differentially expressed (p value < 0.05) (**Figure 6B**). Kyoto Encyclopaedia of Genes and Genomes (KEGG) pathway enrichment analysis showed that these genes were mainly categorized as follows: metabolism, biosynthesis, cell cycle, DNA replication, chemical carcinogenesis, PPAR signalling pathway, and transforming growth factor-β (TGF-β) signalling pathway (**Figure 6A**). A review of the literature showed that the PPAR signalling pathway could play a protective role in both cardiac fibrosis and hepatic fibrosis, suggesting that it may similarly regulate skin fibrosis through the uPA system (24). PPARs include three transcription factors: PPARα, PPARβ, and PPARγ (25). To explore which of these factors may be active in skin fibrosis, their expression patterns were determined in fibroblasts with or without uPAR knockdown. The PPARγ mRNA level was found to be decreased after uPAR knockdown (**Figure 6C**). Subsequently, we treated the uPAR-knockdown fibroblasts with the specific PPARγ agonist pioglitazone and found that the treatment reversed the increased expression of multiple fibrotic factors (**Figure 6D**). Cell migration experiments also showed that the presence of PPARγ could decelerate the migration of fibroblasts, which is conducive to the regression of fibrosis (**Figure 6F**). Since PPARγ was reported to increase the expression of Smad7 in the TGF-β signalling pathway and thus alleviate skin fibrosis, we examined the expression of Smad7 in control fibroblasts, uPAR-knockdown fibroblasts and uPAR-knockdown fibroblasts with an additional PPAR agonist. The results showed that the expression of Smad7 was decreased in the uPAR-siRNA group, and this effect could be reversed by adding a PPAR agonist (**Figure 6E**) (26). These findings suggest that the uPA system is an important signalling pathway in alleviating skin fibrogenesis through the PPAR/Smad7 pathway (**Supplementary Figure 1**).

## Discussion

During the process of physiological repair, various tissue components needs to be dynamically coordinated through all stages of wound healing, which involves changes of soluble mediators, blood cells, ECM, and parenchymal cells (27). In the context of tissue repair, fibrosis contributes to the defence and rapid healing of wounds. However, pathological fibrosis caused by various factors manifests as accumulation of large number of fibroblasts, excessive deposition of ECM, structural destruction of tissues and organs, and continuous loss of function. So far there is no effective treatment for pathological fibrosis, largely due to a poor understanding on its regulatory mechanism.

The characteristic ECM accumulation in fibrosis underscore the importance of proteolytic enzymes that can degrade ECM. Serine proteinases represent an enzyme family most critical for ECM remodelling and degrading of many proteins such as fibrin, fibronectin, and laminin (28).As one of the major system of serine protease systems, uPA system has been proved to be correlated with the degradation of ECM in various malignant tumors. It is commonly accepted that tumour cells and the surrounding stromal cells can produce and release proteolytic enzymes, leading to degradation or disruption of the ECM, thereby promoting tumour invasion and metastasis (29). Accordingly, the function of the uPA system in the degradation of ECM was also studied in fibrotic diseases. It has been observed that the components of uPA system are elevated in a variety of fibrotic diseases including lung fibrosis, renal fibrosis and liver fibrosis, which provide a clinical relevance for the current study on skin fibrosis. Also, Tuan et al. found that PAI-1 may account for the reason of collagen accumulation in keloid fibroblasts using a 3-dimensional fibrin gel model system. And their subsequent study further confirmed the conclusion with adenoviral overexpression and siRNA suppression (30, 31). Nevertheless, the above studies only stay at the molecular level, and they didn’t explore the relevant mechanisms. In this study, we concentrated on the role of the uPA system and its downstream signalling pathway in skin fibrosis. uPA, uPAR and PAI-1 were confirmed to be upregulated in all three kinds of fibrotic skin diseases, indicating the close correlation of the uPA system and skin fibrosis. However, the simultaneous upregulation of uPA and its inhibitor PAI-1 and the opposite effects of the two raised a concern on how the seemingly contradictory changes could coexist and which one may play a dominant role for pathological fibrosis. This question prompted us to determine their individual functions in cell culture and animal models. *In vitro* experiments indicated that knocking down uPAR markedly inhibited myofibroblast migration and promoted myofibroblast proliferation and contraction. This finding demonstrated that uPAR deficiency can accelerate skin fibrosis, which is consistent with the observation of Zhang et al. on renal fibrosis (5). The PAI-1 knockdown experiments showed the opposite effects: the inhibition of cell migration, proliferation and contraction. We next conducted *in vivo* experiments to further investigate the relationship of the uPA system and skin fibrosis and found that uPA binds with uPAR and inhibits fibrosis, while the PAI-1 can promote fibrosis. Mechanistically, proteomics analysis and follow-up experiments showed that the binding of uPA and uPAR activated the downstream PPAR/Smad7 signalling pathway to alleviate skin fibrosis. Noteworthily, when exploring the molecular mechanisms underlying the regulatory effect of the uPA system by proteomic analysis, we found not only PPAR signalling pathway but also TGF-β signalling pathway play a critical role. As a classical signaling pathway, the TGF-β signalling pathway has been proved to be one of the primary factor that drives fibrosis (32). Studies have found that the persistent induction and activation of TGF-β is typically connected with fibrosis, and Rice et al. demonstrated that TGF-β neutralization could decrease fibrosis-associated biomarkers in patients with systemic sclerosis (33, 34). Therefore, in our study we mainly focused on the PPAR signalling pathway. Taken together, these results revealed a novel mechanism of human skin fibrosis and paved the way for new therapeutic strategies.

The bleomycin-induced skin fibrosis mouse model was used to evaluate different therapeutic strategies. It was surprising to found that the local inhibition of PAI-1 or the addition of uPA can both attenuate dermal thickening and collagen deposition. Therefore, we feel that although the expression of uPA and PAI-1 increase at the same time, as long as a sufficient amount of uPA is given to neutralize the effect of PAI-1, the additional part would still play an anti-fibrotic effect. One explanation could be that the increase of uPA represents a feedback or tissue response against the overexpression of PAI-1. Indeed, in most organ systems, increased levels of PAI-1 have been shown to promote the process of fibrosis. It is noteworthy that studies in cardiac fibrosis generated exceptional results. Flevaris et al. observed that PAI-1 could protect against cardiac fibrosis by regulating cardiomyocyte-derived fibrogenic signals and cardiac transcriptional pathways (35). This contradictory observation and its increased levels in skin fibrosis remind us to be precautious about exact role of PAI-1 for skin fibrosis. Further investigation and data are required to reach a reasonable conclusion. In the development of skin fibrosis, various types of cells play a synergistic role. Except the fibroblast that our study mainly focused on, there are many cells influence the whole process. Immune cells are repeatedly reported to be involved in skin fibrosis. Studies have shown that monocytes and macrophages can not only directly produce TGF-β1 that facilitate repair and drive fibrosis, but also control local inflammatory reactions to orchestrate fibrosis (36). Laurent et al. found that Innate lymphoid cells-2 is involved in the development of be involved in the development of lung or hepatic fibrosis through TGFβ pathway (37). With several kinds of immune cells related to TGFβ pathway and fibrosis, we believe it’s highly possible that immune cells play significant roles in uPA-mediated skin fibrosis and our next research will be carried out accordingly.

In summary, we performed a series of *in vitro* and *in vivo* experiments, and the results showed that the uPA system mediates skin fibrosis through the regulation of uPA and PAI-1.Our data indicate that the expression of uPA, uPAR and PAI-1 markedly affects the proliferation, migration, and contractility of fibroblasts and the PPAR signalling pathway may be responsible for the mediating effects of the uPA system on fibroblasts and the process of fibrosis. We hope the novel regulatory mechanism may assist in the development of potential therapeutic targets for skin fibrosis in the future.

## Materials and Methods

### Patients and Ethics Statement

All tissue specimens were collected from patients who underwent surgical excision of hypertrophic scars and keloids and SSc patients who were consistent with the criteria developed by the American College of Rheumatology (ACR) and underwent skin biopsy (**Supplementary Table 1**). The study was performed with approval from the Ethics Committee of Jiangnan University. All of the procedures were explained to the patients, and written informed consent was provided before inclusion. All samples were obtained from tissue that would have been otherwise discarded.

### Cell Isolation and Culture

Fibroblasts were derived from pathological hypertrophic scar tissues collected during scar-removal surgery with the consent of the patients. After fat, blood vessels and fibrous tissue were removed, the tissues were rinsed 2–3 times with PBS, shredded and incubated with collagenase type I (0.1 mg/mL, Sigma) at 37°C for 5 h. The isolated fibroblasts were collected and cultured in medium (DMEM with 10% FBS, penicillin, and streptomycin) at 37°C in 5% CO_2_. Cells were passaged 2–5 times for use in the experiments.

### siRNA Transfection

The chemically synthesized siRNA used in this study was purchased from Sangon Biotech (Shanghai, China), and the transfection reagent Lipofectamine RNAiMAX was purchased from Invitrogen (Carlsbad, USA). Cells were seeded in 6-well plates, 24-well plates, or 96-well plates at the density indicated in the instructions. When 60–70% confluence was reached, siRNAs were transfected into the cells at a final concentration of 40 nM.Interference efficiency was evaluated by real-time quantitative PCR at 48 h and western blot analysis at 24, 48 and 72 h. After the transfection efficiency was confirmed, the cells were used in subsequent experiments.

### Western Blotting

Total proteins were extracted from tissues or fibroblasts using a Total Protein Extraction Kit (Sangon) according to the manufacturer’s instructions, and then the protein concentration was measured with an Enhanced BCA Protein Assay Kit (Beyotime). After the samples were denatured at 100°C for 10 min, equal amounts of protein extract were subjected to electrophoresis in 10% SDS-PAGE gels and then transferred to PVDF membranes (Biosharp). The membranes were blocked with 5% nonfat milk in PBS at room temperature for 1 h and then incubated overnight at 4°C with primary antibodies. Subsequently, the membranes were incubated with horseradish peroxidase-conjugated secondary antibodies for 1 h, and signals were detected using ECL western blotting Substrate (Tanon). The antibodies used were as follows: anti-uPA (PA5-34638, Invitrogen), anti-uPAR (ab103791, ab221680, Abcam), anti-PAI-1 (ab66705, Abcam), anti-Collagen I (ab260043, Abcam; bs-10423, Bioss), anti-α-SMA (ab124964, Abcam; 19245, Cell Signaling), anti-GAPDH (ab181602, Abcam), anti-SMAD1+SMAD5 polyclonal(bs-2973R, Bioss), anti-rabbit IgG (ab150077, Abcam), anti-phospho-SMAD1+SMAD5(Ser463+Ser465) polyclonal antibody (bs-3418R, Bioss), Anti-MADH7/SMAD7(Abcam, ab216428), Anti-PPAR Gamma (ser112) Polyclonal Antibody (Bioss, bs-3737R), anti-rabbit IgG (ab150077, Abcam), and anti-rabbit IgG (8889, Cell Signaling).

### Immunofluorescence Staining

Fibroblasts were fixed with 4% paraformaldehyde for 15 min, permeabilized with 0.5% Triton X-100 for 20 min, and blocked with Immunol Staining Blocking Buffer (Beyotime) for 1 h to block nonspecific binding. Then, the cells were incubated with primary antibodies overnight at 4°C, washed, and incubated with the respective secondary antibodies for 1 h at room temperature. The cells were incubated with DAPI for 10 min at room temperature. Finally, the stained cells were examined using an Olympus DP73 inverted photomicroscope (Olympus, Beijing, China).

### Collagen Gel Contraction Assay

Rat tail tendon collagen type I was purchased from Solarbio Life Sciences and added to 24-well plates according to the instructions of the user guide. The three groups of cells were inoculated in the collagen mixture and placed at room temperature for solidification. After 24 h and 48 h of culture, gel shrinkage was observed and measured.

### Wound−Healing Assay

Cells were plated at a density of 1×10^5^/well in 6-well plates. When the transfected cells reached 100% confluence, a sterile micropipette tip was used to create a scratch. the cells were washed with PBS, and minimal medium was added. Wound healing was observed and imaged at 0 h and after incubation for 6 h and 12 h at 37°C. Cell migration was analysed using ImageJ software.

### Animal Studies

6-Week-old mice were purchased from the Hushan Experimental Animal Centre (Wuxi, China). The animal experiments were approved by the Experimental Animal Committee of Jiangnan University. Mice received subcutaneous injections of 100 μL of 0.5 mg/mL bleomycin dissolved in PBS in a single location on the upper back every other day for 3 weeks to induce dermal fibrosis. Mice that were injected with equal volumes of PBS served as controls. After 3 weeks, the mice were sacrificed by cervical dislocation to evaluate the success of the mouse models. After the establishment of skin fibrosis, the mice were treated with subcutaneous injections of the uPAR inhibitor Amiloride (2 mg/kg/day, MedChemExpress), the uPAR agonist U-Plasminogen Activator Protein (1 mg/kg/day,MedChemExpress) or the PAI-1 inhibitor Tiplaxtinin (5mg/kg, MedChemExpress) daily for an additional 3 weeks (n = 5). On day 42, skin tissues were collected from mice under aseptic conditions and processed for hematoxylin and eosin (HE) staining, Masson’s trichrome staining, immunohistochemistry and the detection of mRNA expression of related genes.

### Statistics

Data are presented as means ± SEM. Multiple groups of samples were analysed by one-way ANOVA, and pairwise comparisons were made by the Bonferroni method. A P value < 0.05 was considered statistically significant. Statistical analyses were performed using GraphPad Prism.

## Data Availability Statement

The original contributions presented in the study are included in the article/**Supplementary Material**. Further inquiries can be directed to the corresponding authors.

## Ethics Statement

The studies involving human participants were reviewed and approved by Ethics Committee of Jiangnan University. The patients/participants provided their written informed consent to participate in this study. The animal study was reviewed and approved by Ethics Committee of Jiangnan University.

## Author Contributions

F-LY and X-JZ designed the whole study, reviewed and revised the manuscript. M-LZ, Y-YT, S-YL, YJ, and Z-DY performed the experiments and wrote the manuscript. K-WZ, Z-HC, J-JW, and X-YT edited and revised the manuscript. SY and J-XY provided patients’ samples and clinical data and interpreted the data. F-LY and XL critically reviewed the whole manuscript. All authors contributed to the article and approved the submitted version.

## Funding

Funding for this study was provided by the Natural Science Foundation of China (81770876) and Natural Science Foundation of Jiangsu Province (Grant BK20191141). Top Talent Support Program for young and middle-aged people of Wuxi Health Committee (BJ2020044: BJ2020057: HB2020043). Fundamental Research Funds of Health and Family Planning Commission of Wuxi (M202024).

## Conflict of Interest

The authors declare that the research was conducted in the absence of any commercial or financial relationships that could be construed as a potential conflict of interest.

## Publisher’s Note

All claims expressed in this article are solely those of the authors and do not necessarily represent those of their affiliated organizations, or those of the publisher, the editors and the reviewers. Any product that may be evaluated in this article, or claim that may be made by its manufacturer, is not guaranteed or endorsed by the publisher.

## References

[1] Weiskirchen R Weiskirchen S Tacke F. Organ and Tissue Fibrosis: Molecular Signals, Cellular Mechanisms and Translational Implications. Mol Aspects Med (2019) 65:2–15. doi: 10.1016/j.mam.2018.06.003 29958900

[2] Henderson NC Rieder F Wynn TA. Fibrosis: From Mechanisms to Medicines. Nature (2020) 587(7835):555–66. doi: 10.1038/s41586-020-2938-9 PMC8034822 33239795

[3] Arndt S Schmidt J Wacker E Karrer S Bosserhoff AK. Fussel-15, A New Player in Wound Healing, Is Deregulated in Keloid and Localized Scleroderma. Am J Pathol (2011) 178(6):2622–31. doi: 10.1016/j.ajpath.2011.02.009 PMC3124099 21641385

[4] Horowitz JC Tschumperlin DJ Kim KK Osterholzer JJ Subbotina N Ajayi IO. Urokinase Plasminogen Activator Overexpression Reverses Established Lung Fibrosis. Thromb Haemost (2019) 119(12):1968–80. doi: 10.1055/s-0039-1697953 PMC7011401 31705517

[5] Zhang G Kim H Cai X López-Guisa JM Alpers CE Liu Y. Urokinase Receptor Deficiency Accelerates Renal Fibrosis in Obstructive Nephropathy. J Am Soc Nephrol (2003) 14(5):1254–71. doi: 10.1097/01.asn.0000064292.37793.fb 12707394

[6] Ma ZG Lv XD Zhan LL Chen L Zou QY Xiang JQ. Human Urokinase-Type Plasminogen Activator Gene-Modified Bone Marrow-Derived Mesenchymal Stem Cells Attenuate Liver Fibrosis in Rats by Down-Regulating the Wnt Signaling Pathway. World J Gastroenterol (2016) 22(6):2092–103. doi: 10.3748/wjg.v22.i6.2092 PMC4726681 26877613

[7] Mahmood N Mihalcioiu C Rabbani SA. Multifaceted Role of the Urokinase-Type Plasminogen Activator (uPA) and Its Receptor (uPAR): Diagnostic, Prognostic, and Therapeutic Applications. Front Oncol (2018) 8:24. doi: 10.3389/fonc.2018.00024 29484286 PMC5816037

[8] Busso N Masur SK Lazega D Waxman S Ossowski L. Induction of Cell Migration by Pro-Urokinase Binding to its Receptor: Possible Mechanism for Signal Transduction in Human Epithelial Cells. J Cell Biol (1994) 126(1):259–70. doi: 10.1083/jcb.126.1.259 PMC2120093 7517943

[9] Danø K Behrendt N Høyer-Hansen G Johnsen M Lund LR Ploug M. Plasminogen Activation and Cancer. Thromb Haemost (2005) 93(4):676–81. 10.1160/TH05-01-0054 15841311

[10] Vassalli JD Baccino D Belin D. A Cellular Binding Site for the Mr 55,000 Form of the Human Plasminogen Activator, Urokinase. J Cell Biol (1985) 100(1):86–92. doi: 10.1083/jcb.100.1.86 3880760 PMC2113459

[11] Binder BR Mihaly J Prager GW. uPAR-uPA-PAI-1 Interactions and Signaling: A Vascular Biologist’s View. Thromb Haemost (2007) 97(3):336–42. doi: 10.1160/th06-11-0669 17334498

[12] Dinesh P Rasool M. uPA/uPAR Signaling in Rheumatoid Arthritis: Shedding Light on its Mechanism of Action. Pharmacol Res (2018) 134:31–9. doi: 10.1016/j.phrs.2018.05.016 29859810

[13] Malinowsky K Wolff C Berg D Schuster T Walch A Bronger H. uPA and PAI-1-Related Signaling Pathways Differ Between Primary Breast Cancers and Lymph Node Metastases. Transl Oncol (2012) 5(2):98–104. doi: 10.1593/tlo.11268 22496926 PMC3323931

[14] Smith HW Marshall CJ. Regulation of Cell Signalling by uPAR. Nat Rev Mol Cell Biol (2010) 11(1):23–36. doi: 10.1038/nrm2821 20027185

[15] Flevaris P Vaughan D. The Role of Plasminogen Activator Inhibitor Type-1 in Fibrosis. Semin Thromb Hemost (2017) 43(2):169–77. 10.1055/s-0036-1586228 27556351

[16] Ghosh AK Vaughan DE. PAI-1 in Tissue Fibrosis. J Cell Physiol (2012) 227(2):493–507. doi: 10.1002/jcp.22783 21465481 PMC3204398

[17] Wynn TA Ramalingam TR. Mechanisms of Fibrosis: Therapeutic Translation for Fibrotic Disease. Nat Med (2012) 18:1028–40. doi: 10.1038/nm.2807 PMC3405917 22772564

[18] Chang J Lan T Li C Ji X Zheng L Gou H. Activation of Slit2-Robo1 Signaling Promotes Liver Fibrosis. J Hepatol (2015) 63:1413–20. doi: 10.1016/j.jhep.2015.07.033 26264936

[19] Tao L Bei Y Chen P Lei Z Fu S Zhang H. Crucial Role of miR-433 in Regulating Cardiac Fibrosis. Theranostics (2016) 6:2068–83. doi: 10.7150/thno.15007 PMC5039681 27698941

[20] Feng Y Wu JJ Sun ZL Liu SY Zou ML Yuan ZD. Targeted Apoptosis of Myofibroblasts by Elesclomol Inhibits Hypertrophic Scar Formation. EBioMedicine (2020) 54:102715. doi: 10.1016/j.ebiom.2020.102715 32251998 PMC7132150

[21] Adnot S Breau M Houssaini A. PAI-1: A New Target for Controlling Lung-Cell Senescence and Fibrosis. Am J Respir Cell Mol Biol (2020) 62(3):271–2. doi: 10.1165/rcmb.2019-0341ED PMC7055697 31622556

[22] Rabieian R Boshtam M Zareei M Kouhpayeh S Masoudifar A Mirzaei H. Plasminogen Activator Inhibitor Type-1 as a Regulator of Fibrosis. J Cell Biochem (2018) 119(1):17–27. doi: 10.1002/jcb.26146 28520219

[23] Samarakoon R Overstreet JM Higgins SP Higgins PJ. TGF-β1 → SMAD/p53/USF2 → PAI-1 Transcriptional Axis in Ureteral Obstruction-Induced Renal Fibrosis. Cell Tissue Res (2012) 347(1):117–28. doi: 10.1007/s00441-011-1181-y PMC3188682 21638209

[24] Xu M Xu HH Lin Y Sun X Wang LJ Fang ZP. LECT2, a Ligand for Tie1, Plays a Crucial Role in Liver Fibrogenesis. Cell (2019) 178(6):1478–92.e20. doi: 10.1016/j.cell.2019.07.021 31474362

[25] Korbecki J Bobiński R Dutka M. Self-Regulation of the Inflammatory Response by Peroxisome Proliferator-Activated Receptors. Inflamm Res (2019) 68(6):443–58. doi: 10.1007/s00011-019-01231-1 PMC6517359 30927048

[26] Ni XX Li XY Wang Q Hua J. Regulation of Peroxisome Proliferator-Activated Receptor-Gamma Activity Affects the Hepatic Stellate Cell Activation and the Progression of NASH *via* TGF-β1/Smad Signaling Pathway. J Physiol Biochem (2021) 77(1):35–45. doi: 10.1007/s13105-020-00777-7 33188625

[27] Zou ML Teng YY Wu JJ Liu SY Tang XY Jia Y. Fibroblasts: Heterogeneous Cells With Potential in Regenerative Therapy for Scarless Wound Healing. Front Cell Dev Biol (2021) 9:713605. doi: 10.3389/fcell.2021.713605 34354997 PMC8329665

[28] Lu P Takai K Weaver VM Werb Z. Extracellular Matrix Degradation and Remodeling in Development and Disease. Cold Spring Harb Perspect Biol (2011) 3(12). doi: 10.1101/cshperspect.a005058 PMC3225943 21917992

[29] Herszényi L Barabás L Hritz I István G Tulassay Z. Impact of Proteolytic Enzymes in Colorectal Cancer Development and Progression. World J Gastroenterol (2014) 20(37):13246–57. doi: 10.3748/wjg.v20.i37.13246 PMC4188883 25309062

[30] Tuan TL Hwu P Ho W Yiu P Chang R Wysocki A. Adenoviral Overexpression and Small Interfering RNA Suppression Demonstrate That Plasminogen Activator Inhibitor-1 Produces Elevated Collagen Accumulation in Normal and Keloid Fibroblasts. Am J Pathol (2008) 173(5):1311–25. doi: 10.2353/ajpath.2008.080272 PMC2570122 18832570

[31] Tuan TL Zhu JY Sun B Nichter LS Nimni ME Laug WE. Elevated Levels of Plasminogen Activator Inhibitor-1 may Account for the Altered Fibrinolysis by Keloid Fibroblasts. J Invest Dermatol (1996) 106(5):1007–11. doi: 10.1111/1523-1747.ep12338552 8618030

[32] Meng XM Nikolic-Paterson DJ Lan HY. TGF-β: The Master Regulator of Fibrosis. Nat Rev Nephrol (2016) 12(6):325–38. doi: 10.1038/nrneph.2016.48 27108839

[33] Frangogiannis N. Transforming Growth Factor-β in Tissue Fibrosis. J Exp Med (2020) 217(3):e20190103. doi: 10.1084/jem.20190103 32997468 PMC7062524

[34] Rice LM Padilla CM McLaughlin SR Mathes A Ziemek J Goummih S. Fresolimumab Treatment Decreases Biomarkers and Improves Clinical Symptoms in Systemic Sclerosis Patients. J Clin Invest (2015) 125(7):2795–807. doi: 10.1172/JCI77958 PMC4563675 26098215

[35] Flevaris P Khan SS Eren M Schuldt A Shah SJ Lee DC. Plasminogen Activator Inhibitor Type I Controls Cardiomyocyte Transforming Growth Factor-β and Cardiac Fibrosis. Circulation (2017) 136(7):664–79. doi: 10.1161/CIRCULATIONAHA.117.028145 PMC5784400 28588076

[36] Wynn TA Vannella KM. Macrophages in Tissue Repair, Regeneration, and Fibrosis. Immunity (2016) 44(3):450–62. doi: 10.1016/j.immuni.2016.02.015 PMC4794754 26982353

[37] Laurent P Allard B Manicki P Jolivel V Levionnois E Jeljeli M. Tgfβ Promotes Low IL10-Producing ILC2 With Profibrotic Ability Involved in Skin Fibrosis in Systemic Sclerosis. Ann Rheum Dis (2021) 80(12):1594–603. doi: 10.1136/annrheumdis-2020-219748 PMC8600612 34285051

