# Supplementary material for: The uPA System Differentially Alters Fibroblast Fate and Profibrotic Ability in Skin Fibrosis
Source: Front Immunol. 2022 Mar 16;13:845956. doi: 10.3389/fimmu.2022.845956 (PMC8966095; doi:10.3389/fimmu.2022.845956)

Title: The uPA system differentially alters fibroblast fate and profibrotic ability in skin fibrosis

Online supplemental figure 1

uPA-uPAR regulates skin fibrosis through PPAR/Smad7 signalling pathway.

Online supplemental figure 2

Characterization of knocking down uPAR or PAI-1 in disease-derived fibroblasts.

(A) Immunofluorescence staining confirmed the expression of uPA, uPAR and PAI-1 in disease-derived fibroblasts. (B) The knockdown efficiency in different time point of uPAR-siRNA in disease-derived fibroblasts was confirmed by western blot. (C) The knockdown efficiency in different time point of PAI-1-siRNA in disease-derived fibroblasts was confirmed by western blot. (D) Quantification of collagen gel contraction assay on knocking down uPAR. (E) Quantification of collagen gel contraction assay on knocking down PAI-1. (F) Quantification of scratch wound assays on knocking down uPAR. (G) Quantification of scratch wound assays on knocking down PAI-1.

Online supplemental figure 3

(A) The evaluation of the success of the mouse models. (B) Volcano plot for differernt expression of genes in uPAR-siRNA and control group. The blue and red dots indicate the significantly downregulated and upregulated genes. (C) Prediction and analysis of proteins using the enrichments of gene ontology.

Tables:

Supplementary Table 1: The patients' clinical characteristics

Species	Age(years)	Gender	Disease duration (month)	Site	Subtype	mRSS
Hypertrophic scars (n=5)						
H1	46	Male	22	foot		
H2	34	Female	17	shoulder		
H3	26	Female	12	shoulder		
H4	20	Female	9	forearm		
H5	25	Male	10	forearm		
Keloids (n=4)						
K1	39	Male	17	ear		
K2	43	Male	30	forearm		
K3	31	Male	22	forearm		
K4	24	Female	18	chest		
Systemic sclerosis (n=7)						
S1	43	Female	15	forearm	lcSSc	14
S2	38	Female	24	forearm	lcSSc	22
S3	45	Female	21	forearm	lcSSc	15
S4	33	Female	13	forearm	lcSSc	18
S5	39	Male	19	forearm	dcSSc	32
S6	27	Female	20	forearm	dcSSc	10
S7	41	Female	24	forearm	dcSSc	4

dcSSc, diffuse cutaneous SSc; lcSSc, limited cutaneous SSc. dcSSc, diffuse cutaneous SSc; lcSSc, limited cutaneous SSc

Supplementary Table 2: Sequence of siRNA used in this study

Gene	Sequences	
	Sense(5'-3')	Antisense(5'-3')
uPAR-559	GCCGUUACCUCGAAUGCAUTT	AUGCAUUCGAGGUAACGGCTT
uPAR-1048	GUGACGCCUUCAGCAUGAATT	UUCAUGCUGAAGGCGUCACTT
PAI-1-731	GCCUUCAGCAUGAACCACAUUTT	AAUGUGGUUCAUGCUGAAGGCTT
PAI-1-294	GCUUGAAGAUCACCAGCCUUATT	UAAGGCUGGUGAUCUUCAAGCTT
PAI-1-610	CCACUUCCUGAAAUGCUGCAATT	UUGCAGCAUUUCAGGAAGUGGTT
NC	UUCUCCGAACGUGUCACGUTT	ACGUGACACGUUCGGAGAATT
GAPDH	GUAUGACAACAGCCUCAAGTT	CUUGAGGCUGUUGUCAUACTT

Supplementary Table 3 Sequences of primers used for qPCR

**mRNA**	**Primer**	
	**FORWARD**	**REVERSE**
uPAR	GAACAGTGCCTGGATGTGGTGAC	AGGAAGTGGAAGGTGTCGTTGTTG
uPA	TCGCTCAAGGCTTAACTCCAACAC	ACGGATCTTCAGCAAGGCAATGTC
PAI-1	GTGCTGGTGAATGCCCTCTACTTC	TGCTGCCGTCTGATTTGTGGAAG
α-SMA	CTTCGTTACTACTGCTGAGCGTGAG	CCATCAGGCAACTCGTAACTCTTCTC
C0l-1	CAAGAGTGGTGATCGTGGTGAGAC	CTTTATGCCTCTGTCGCCCTGTTC
PPARα	CTGTCTGCTCTTCAGGGAACTGTTAC	GTCTGTGGTTTCTGCTTTCTGCTTTG
PPARβ/δ	TGGTGTGGAAGCAGTTGGTGAATG	GGATGCTCTTGGCGAACTCAGTG
PPARγ	TCTCCAGCATTTCTACTCCACATTACG	CAGGCTCCACTTTGATTGCACTTTG
Ets-1	TCCCACTATTAACTCCAAGCAGCAAAG	CACATCACCCAGTCCCGAACATG
TGFβ	TACAGCAACAATTCCTGGCGATACC	CTCAACCACTGCCGCACAACTC
FGFR1	TGAAGTCGGACGCAACAGAGAAAG	GGCATACTCCACGATGACATACAAGG

Online supplemental figure 1

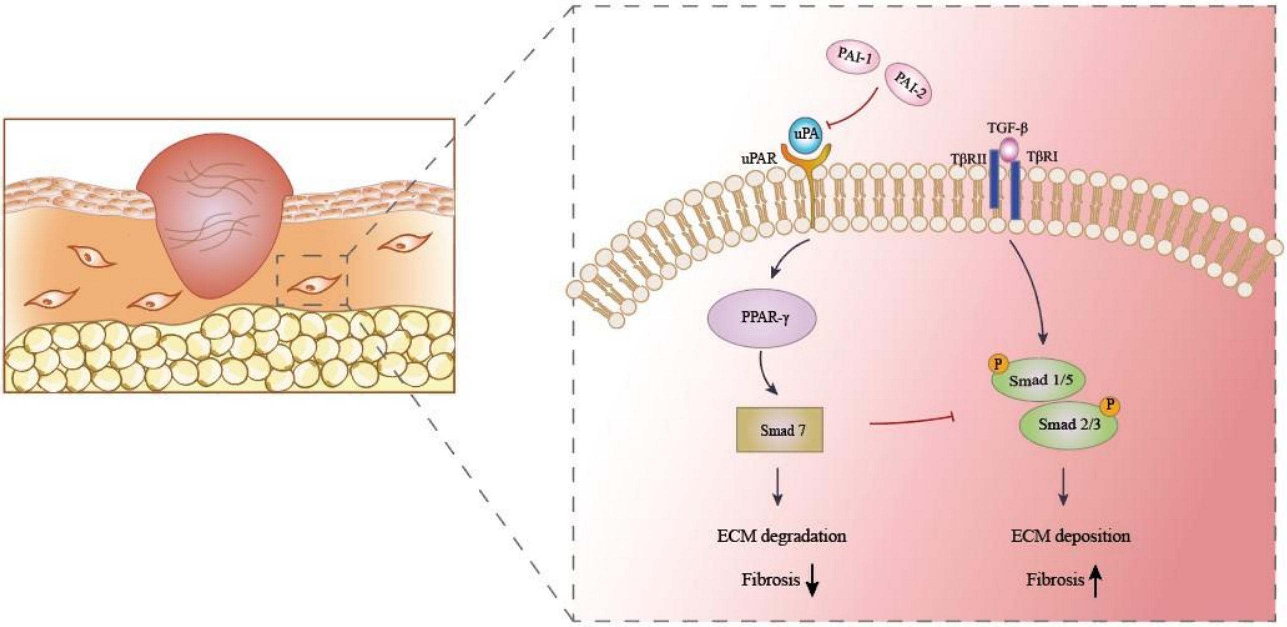

Online supplemental figure 2

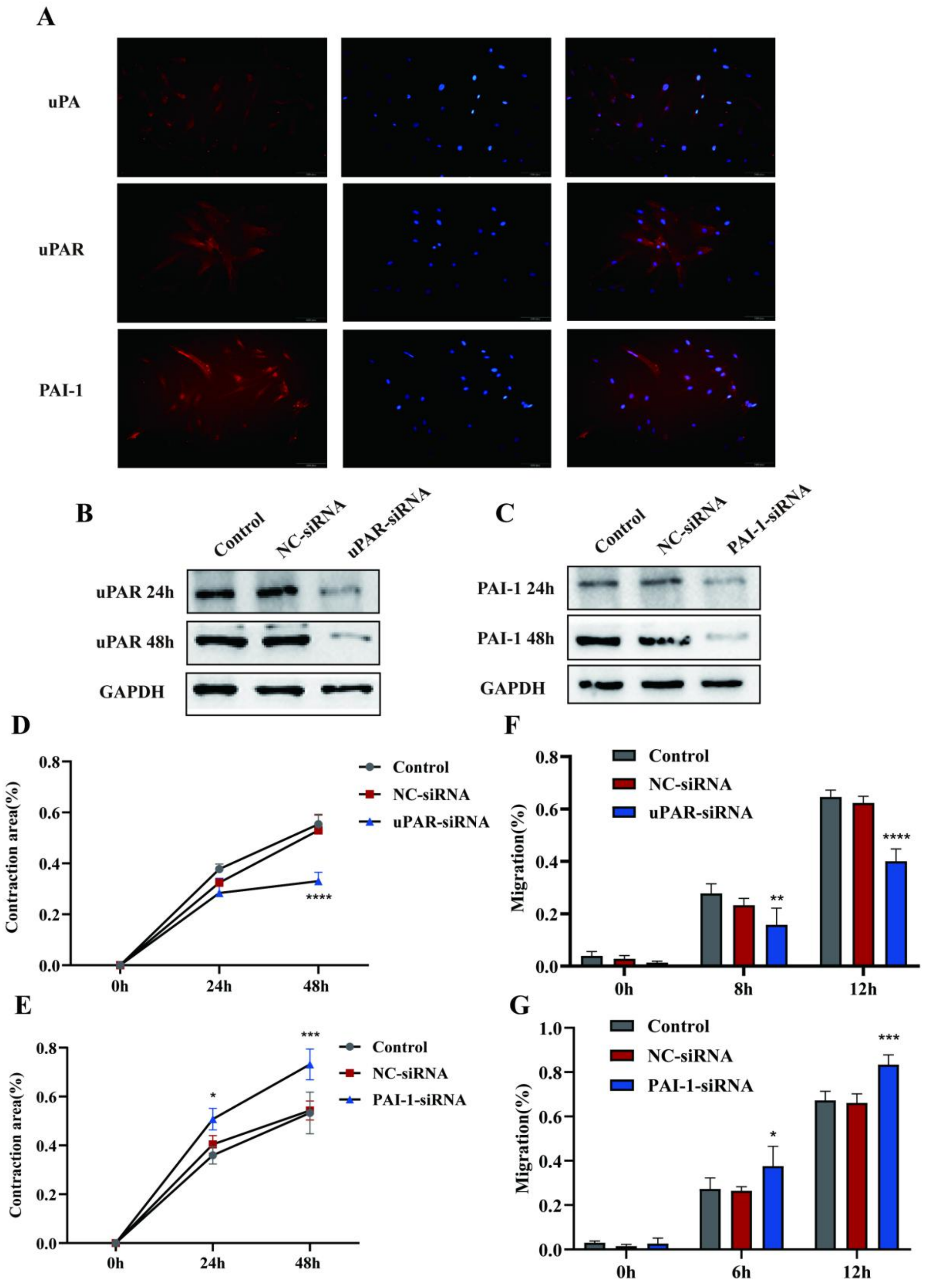

Online supplemental figure 3

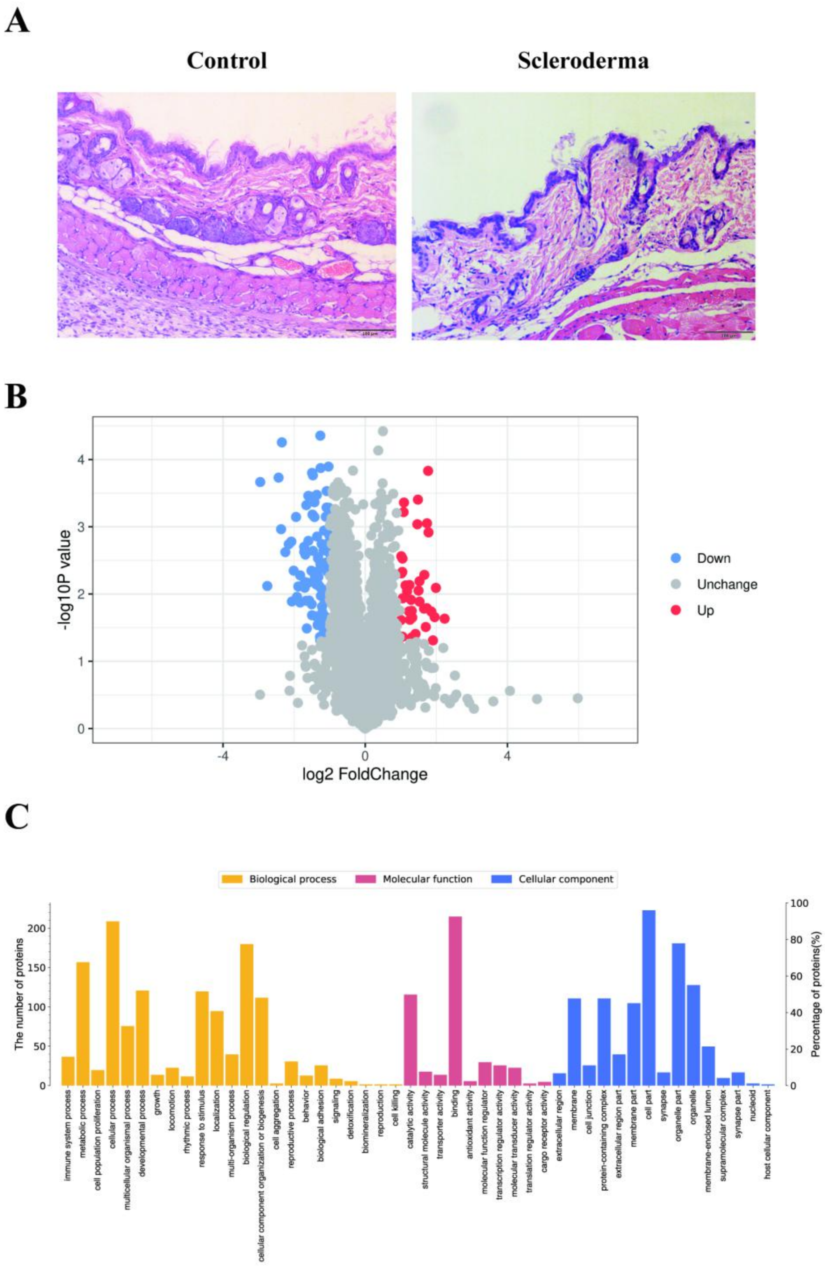

Supplement: Supplementary file 1 [file DataSheet_1.pdf]
